# Effect of transfusion of fresh frozen plasma on parameters of endothelial condition and inflammatory status in non-bleeding critically ill patients: a prospective substudy of a randomized trial

**DOI:** 10.1186/s13054-015-0828-6

**Published:** 2015-04-15

**Authors:** Marleen Straat, Marcella CA Müller, Joost CM Meijers, Mendi S Arbous, Angelique ME Spoelstra - de Man, Charlotte JP Beurskens, Margreeth B Vroom, Nicole P Juffermans

**Affiliations:** Department of Intensive Care Medicine, Academic Medical Center, Meibergdreef 9, Amsterdam, 1105 AZ The Netherlands; Laboratory of Experimental Intensive Care and Anesthesiology, Academic Medical Center, Meibergdreef 9, Amsterdam, 1105 AZ The Netherlands; Department of Experimental Vascular Medicine, Academic Medical Center, Meibergdreef 9, Amsterdam, 1105 AZ The Netherlands; Department of Intensive Care Medicine, Leiden University Medical Center, Albinusdreef 2, Leiden, 2333 ZA The Netherlands; Department of Intensive Care Medicine, VU Medical Center, De Boelelaan 1117, Amsterdam, 1081 HZ The Netherlands

## Abstract

**Introduction:**

Much controversy exists on the effect of a fresh frozen plasma (FFP) transfusion on systemic inflammation and endothelial damage. Adverse effects of FFP have been well described, including acute lung injury. However, it is also suggested that a higher amount of FFP decreases mortality in trauma patients requiring a massive transfusion. Furthermore, FFP has an endothelial stabilizing effect in experimental models. We investigated the effect of fresh frozen plasma transfusion on systemic inflammation and endothelial condition.

**Methods:**

A prospective predefined substudy of a randomized trial in coagulopathic non-bleeding critically ill patients receiving a prophylactic transfusion of FFP (12 ml/kg) prior to an invasive procedure. Levels of inflammatory cytokines and markers of endothelial condition were measured in paired samples of 33 patients before and after transfusion. The statistical tests used were paired *t* test or the Wilcoxon signed-rank test.

**Results:**

At baseline, systemic cytokine levels were mildly elevated in critically ill patients. FFP transfusion resulted in a decrease of levels of TNF-α (from 11.3 to 2.3 pg/ml, *P* = 0.01). Other cytokines were not affected. FFP also resulted in a decrease in systemic syndecan-1 levels (from 675 to 565 pg/ml, *P* = 0.01) and a decrease in factor VIII levels (from 246 to 246%, *P* <0.01), suggestive of an improved endothelial condition. This was associated with an increase in ADAMTS13 levels (from 24 to 32%, *P* <0.01) and a concomitant decrease in von Willebrand factor (vWF) levels (from 474 to 423%, *P* <0.01).

**Conclusions:**

A fixed dose of FFP transfusion in critically ill patients decreases syndecan-1 and factor VIII levels, suggesting a stabilized endothelial condition, possibly by increasing ADAMTS13, which is capable of cleaving vWF.

**Trial registrations:**

Trialregister.nl NTR2262, registered 26 March 2010 and Clinicaltrials.gov NCT01143909, registered 14 June 2010.

## Introduction

Substantial units of fresh frozen plasma (FFP) are utilized in the intensive care unit (ICU) [1,2]. FFP is effective in correcting clotting factor deficiencies [3] and is therefore transfused in patients with active bleeding, but also frequently in patients with abnormal coagulation tests to prevent bleeding [2,4]. In sepsis patients, FFP transfusion rates of up to 57% have been reported [5]. However, there is an association between FFP transfusion and adverse outcome in the critically ill, including transfusion-related acute lung injury (TRALI) [4,6-8], transfusion-related circulatory overload [9,10], multiorgan failure [8,11] and an increased risk of infections [12]. Although not entirely understood, the pathological mechanisms underlying the association between FFP transfusion and lung injury is thought to result from an inflammatory response including a neutrophil influx into the lungs and elevated pulmonary levels of interleukin 8 (IL-8) and interleukin 1 (IL-1), as demonstrated in TRALI patients [13,14]. In line with this, FFP increased expression of endothelial adhesion molecules in human pulmonary endothelial cells [15]. Together, these data suggest that endothelial cell activation and disruption may be an early event following lung injury due to transfusion [16].

On the other hand, FFP also seems to have protective effects. In trauma patients requiring a massive transfusion, resuscitation with a higher ratio of FFP to red blood cell units is associated with decreased mortality [17,18]. Interestingly, some studies suggest that this decreased mortality is irrespective of correction of coagulopathy by restoring coagulation factor levels [18,19], although not all studies support this observation [20,21]. Instead, a beneficial effect of FFP may be related to the restoration of injured endothelium. Syndecan-1 is a proteoglycan on the luminal surface of endothelial cells that inhibits leukocyte adhesion. During endothelial damage, syndecan-1 is shed, resulting in increased levels of syndecan-1 in the systemic compartment [22]. Patients in hemorrhagic shock have a disrupted endothelial integrity and glycocalyx layer, with decreased syndecan-1 expression [23]. Vascular integrity is also disrupted in various populations of critically ill patients, as demonstrated by increased systemic levels of syndecan-1 [24,25]. Of interest, in a hemorrhagic shock model, FFP was found to improve endothelial integrity, associated with increased expression of syndecan-1 on endothelial cells [26].

The effect of transfusion of FFP on endothelial and cytokine host response in patients is unknown. In a study investigating the risk-benefit ratio of FFP transfusion in non-bleeding critically ill patients with a coagulopathy, we investigated the inflammatory and endothelial host response to a fixed dose of FFP transfusion.

## Methods

### Study design

This was a predefined *post hoc* substudy of a multicenter trial in which non-bleeding critically ill patients with an increased international normalized ratio (INR, 1.5 to 3.0) were randomized between May 2010 and June 2013 to omitting or administering a prophylactic transfusion of FFP (12 ml/kg) prior to an invasive procedure. Only patients randomized to receive FFP were included in this substudy. Patients were enrolled at three sites in The Netherlands: two university hospitals (Academic Medical Center, Amsterdam and Leiden University Medical Center, Leiden) and one large teaching hospital (Tergooi Ziekenhuizen, Hilversum). The Institutional Review Board of the Academic Medical Center approved the study protocol. Before entry in the study, written informed consent was obtained from the patient or legal representative in accordance with the Declaration of Helsinki. The study protocol was registered with trial identification numbers NTR2262 and NCT01143909 [27].

Exclusion criteria were clinically overt bleeding, thrombocytopenia of <30 × 10^9^/L, treatment with vitamin K antagonists, activated protein C, abciximab, tirofiban, ticlopidine or prothrombin complex concentrates and a history of congenital or acquired coagulation factor deficiency or bleeding diathesis. Patients treated with low-molecular-weight heparin (LMWH) or heparin in therapeutic dose were eligible if medication was discontinued for an appropriate period. Sepsis was defined by the Bone criteria [28]. Disseminated intravascular coagulation (DIC) was defined by an International Society on Thrombosis and Haemostasis (ISTH) score of ≥5 [29]. The FFP was quarantine plasma manufactured by Sanquin, the Dutch National Blood Bank. As of 2007, women are deferred for donation for preparation of FFP in the Netherlands.

### Sample collection

Citrated blood samples were drawn from an indwelling arterial catheter before and within 10 minutes after FFP transfusion. During transfusion, respiratory settings were kept constant. Samples were collected in sodium citrate (0.109 M 3.2%) tubes and were centrifuged twice within 30 minutes: the first 15 minutes at 2,000 × *g* and then 5 minutes at 15,000 × *g*, both at 18°C. Supernatant was collected and stored at −80°C.

### Assays

Tumor necrosis factor alpha (TNF-α) levels were measured by enzyme-linked immunosorbent assay (ELISA), according to instructions of the manufacturer (R&D Systems Inc., Minneapolis, MN, USA). Serum levels of interleukin 1 beta (IL-1β), interleukin 1 receptor antagonist (IL-1RA), IL-8, interleukin 10 (IL-10), macrophage inflammatory proteins (MIP)-1A, monocyte chemotactic protein (MCP)-1 and soluble CD40 ligand (sCD40L) were determined by Luminex, according to the manufacturer’s instructions (Merck Millipore Chemicals BV, Amsterdam, The Netherlands). When less than 50 beads were measured by the Luminex assay, samples were excluded from further analysis. von Willebrand factor antigen (vWF:Ag) levels were determined with an in-house ELISA using commercially available polyclonal antibodies against von Willebrand factor (vWF) (Dako, Glostrup, Denmark). ADAMTS13 (a disintegrin and metalloproteinase with a thrombospondin type 1 motif, member 13) activity was determined as described earlier [30]. Factor VIII activity was determined on a Behring XP coagulation analyzer using reagents and protocols from the manufacturer (Siemens Healthcare Diagnostics, Marburg, Germany).

### Statistical analysis

Variables are expressed as medians and interquartile ranges (IQRs) or means and standard deviations (SDs). For comparisons, a paired *t* test was used, or the Wilcoxon signed-rank test in case of not normally distributed data. For the analyses, we used SPSS version 21.0 (IBM Corp., Armonk, NY, USA) and Graphpad Prism 5 (Graphpad Software Inc., San Diego, CA, USA).

## Results

### Patients

From 38 patients receiving FFP, paired samples from 33 patients were available for analysis before and after FFP transfusion. Patients were ill, as reflected by a high disease severity score and half of the patients had sepsis (Table 1). Patients received a mean dosage of 11.2 (2.8) ml/kg FFP, which was transfused in 121 ± 43 minutes.

**Table 1:** **Patient characteristics**

	**FFP transfusion N = 33**
**General characteristics**	
Gender, male, n (%)	21 (64)
Age (years)	61 (50–70)
APACHE IV score	96 (79–128)
SOFA score	11 (10–14)
**Medical history**	
Pulmonary disease, n (%)	3 (9)
Liver disease, n (%)	6 (18)
Cardiac failure, n (%)	6 (18)
**Medical condition 24 hours before transfusion**	
Mechanical ventilation, n (%)	27 (82)
Sepsis, n (%)	15 (45)
Disseminated intravascular coagulation, n (%)	16 (49)
**Clinical outcomes**	
ICU length of stay	12 (6–23)
Mortality	21 (64)

Data expressed as median and interquartile ranges. FFP, fresh frozen plasma; APACHE IV score, acute physiology and chronic health evaluation IV score; SOFA score, sequential organ failure assessment score; ICU, intensive care unit.

### Inflammatory cytokine and chemokine levels before and after transfusion of 12 ml/kg FFP

At baseline, levels of cytokines were mildly elevated in this cohort. After FFP transfusion, median TNF-α decreased (*P* = 0.01, Table 2). Levels of all other cytokines were not affected by FFP transfusion. Chemokine levels IL-8 and MCP-1 were elevated at baseline but also not influenced by FFP transfusion. Levels of sCD40L, which has been implicated as a mediator in TRALI [31], were also not significantly altered by FFP transfusion.

**Table 2:** **Inflammatory cytokines in critically ill patients before and after a transfusion of fresh frozen plasma (12 ml/kg)**

	**Before FFP**	**After FFP**	***P*** **value**
***Proinflammatory parameters (pg/ml)***			
TNF-α	11.3 (2.3–52.3)	2.3 (2.3–41.0)	0.01
IL-1β	15.0 (11.7–18.8)	14.4 (13.1–23.3)	0.97
IL-8	178 (124–418)	187 (113–412)	0.23
MCP-1	1255 (503–3376)	1101 (434–5802)	0.89
MIP-1A	19.6 (15.7–33.6)	19.1 (13.3–34.4)	0.12
sCD40L	409 (257–614)	324 (216–537)	0.08
***Anti-inflammatory parameters (pg/ml)***			
IL-1RA	69.3 (52.1–110.6)	73.5 (47.8–104.9)	0.11
IL-10	36.1 (15.5–100.1)	31.5 (14.8–279.6)	0.62

Data expressed as median (IQR). FFP, fresh frozen plasma; TNF-α, tumor necrosis factor alpha; IL-1β, interleukin 1 beta; IL-8, interleukin 8; MCP-1, monocyte chemotactic protein 1; MIP-1A, macrophage inflammatory proteins 1A; sCD40L, soluble CD40 ligand; IL-1RA, interleukin 1 receptor antagonist; IL-10, interleukin 10; IQR, interquartile range.

### Parameters of endothelial condition before and after transfusion of 12 ml/kg FFP

**Figure 1:**
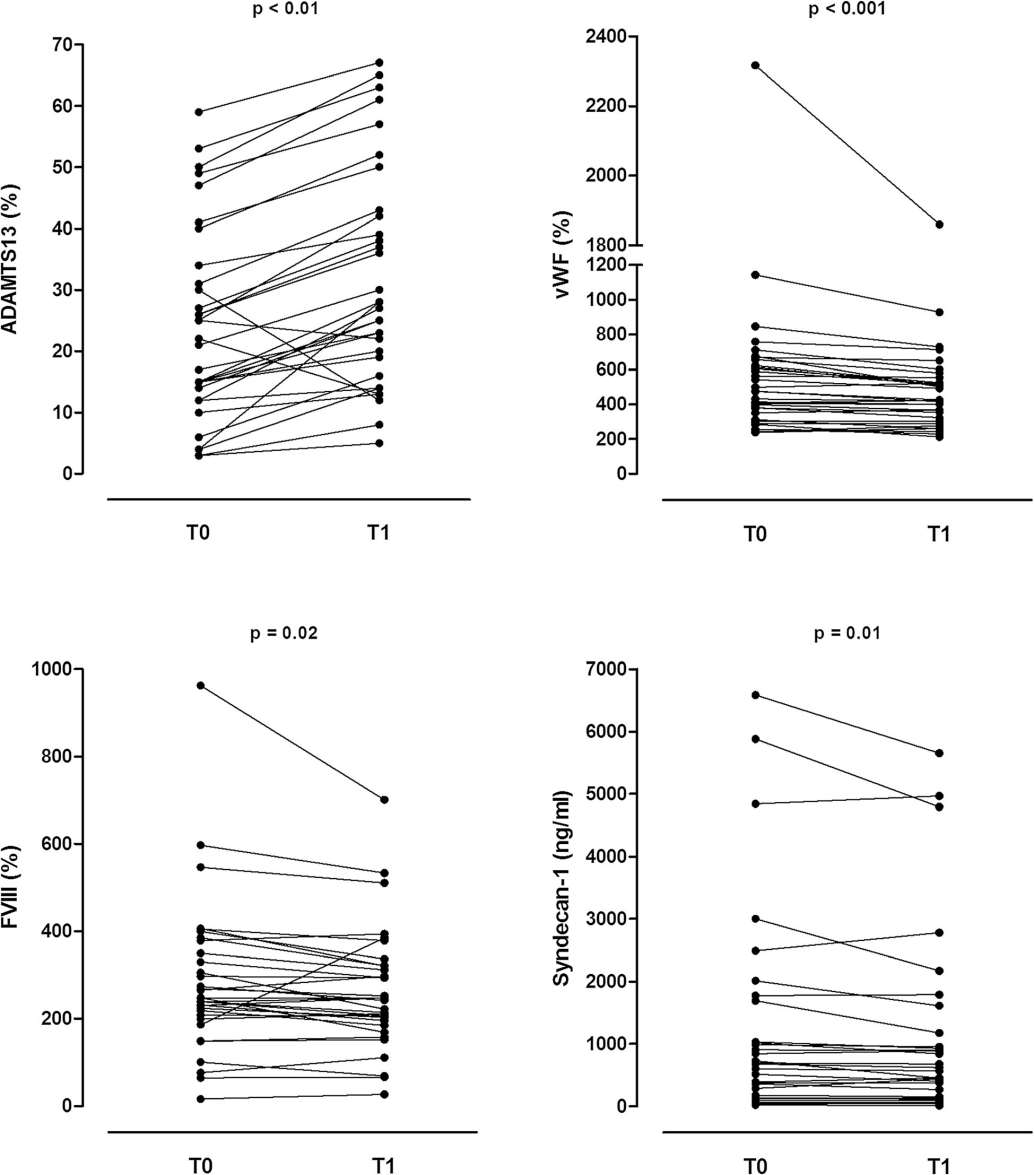
**Markers of endothelial condition in critically ill patients before and after a transfusion of fresh frozen plasma (12 ml/kg): ADAMTS13, von Willebrand factor, factor VIII and syndecan-1.** ADAMTS13, a disintegrin and metalloproteinase with a thrombospondin type 1 motif, member 13.

After FFP transfusion, levels of ADAMTS13 increased (*P* <0.01, Table 3 and Figure 1). This increase was accompanied by a decrease in vWF (*P* <0.001) and in systemic levels of syndecan-1 (*P* = 0.01). Factor VIII levels were slightly decreased following FFP transfusion (*P* = 0.02).

**Table 3:** **Parameters of endothelial condition in critically ill patients before and after a transfusion of fresh frozen plasma (12 ml/kg)**

	**Before FFP**	**After FFP**	***P*** **value**
vWF-Ag	474.0 (331.5–639.5)	423.0 (313.5–539.0)	<0.001
Factor VIII	246.4 (203.6–364.4)	246.40 (321.2)	<0.01
ADAMTS13	23.9 (15.8)	31.7 (17.9)	<0.001
Syndecan-1	674.6 (132.2–1689.8)	565.1 (126.8–1175.7)	<0.01

Data expressed as median (IQR) or mean (SD). FFP, fresh frozen plasma; vWF:Ag, von Willebrand factor antigen; ADAMTS13, a disintegrin and metalloproteinase with a thrombospondin type 1 motif, member 13; IQR, interquartile range; SD, standard deviation.

## Discussion

Our patients had mildly elevated levels of inflammatory cytokines at baseline, which corresponds to levels measured before in critically ill patients [32]. We observed no aggravation of this inflammatory response after FFP transfusion. Rather, there was a decrease in TNF-α level. This is not in line with a study in which FFP elicited an inflammatory response in endothelial cells [33], nor with an *in vitro* model of transfusion, in which whole blood incubated with FFP induced TNF-α production [34].

Of the cytokines we measured, only TNF-α changed after FFP transfusion. As TNF-α is known to be the quickest responder among all cytokines, we may have timed our measurement too early after FFP transfusion to note an effect of FFP on other cytokine levels. However, lung injury following transfusion is thought to be an early event. Also, we choose this early time point to minimize confounding by other factors. Taken together, FFP does not appear to elicit an early inflammatory response.

Of interest, recent *in vitro* studies support an endothelial stabilizing role of FFP, as FFP reduced vascular endothelial cell permeability [26,35] and decreased expression of endothelial adhesion markers [36] and endothelial white blood cell binding [26,36,37]. Effects of FFP were investigated in a rat hemorrhagic shock model, characterized by systemic shedding of syndecan-1, decreased syndecan-1 expression on pulmonary cells and increased pulmonary vascular permeability. Resuscitation with FFP abrogated these effects, whereas resuscitation with crystalloids did not [26], and was associated with preservation of the endothelial glycocalyx [38] and improvement of lung injury [39].

In trauma patients with hemorrhagic shock, syndecan-1 levels are also increased [23]. Studies of the effect of FFP on endothelial condition in patients are, however, lacking. Of note, recent evidence in trauma patients requiring a massive transfusion suggests that higher dose and earlier administration of FFP decreases mortality [17,18,40,41]. This effect was not associated with improved coagulation ability, as the reduction in mortality in their study was irrespective of the admission INR [18] and coagulopathy does not seem to improve with higher amounts of FFP [19]. Given that FFP restores coagulation factors but also anti-coagulant proteins and that the net effect on hemostasis is unclear, FFP may exert protection via other mechanisms. We found that FFP decreased levels of syndecan-1, associated with decreased levels of factor VIII, which both reflect improved endothelial condition. These results support earlier experimental work indicating that FFP preserves endothelial integrity.

The mechanism underlying this beneficial effect of FFP has not yet been described. We found that FFP transfusion was associated with an increase in ADAMTS13 and a decrease in vWF. Thereby, ADAMTS13 may have increased the ability to cleave large vWF multimers present on the activated endothelium. As large vWF multimers damage the endothelium, this effect may have preserved endothelial condition. This is also the rationale behind the treatment of thrombotic thrombocytopenic purpura by therapeutic plasma exchange.

A protective effect of FFP on the endothelium is in apparent contrast with studies that have linked FFP to the occurrence of TRALI [4,6-8]. In an effort to reconcile these findings, we suggest that FFP associated with TRALI occurs as a result of an antibody-mediated pathogenesis. Indeed, efforts to reduce antibody-positive blood products by male-only policies are associated with a significant reduction in TRALI [42]. In patients in whom transfusion is associated with lung injury in the absence of antibodies, other products such as red blood cells and platelets may be more important in inducing lung injury. Although dissecting these effects in multiple transfused patients is a challenge, future research should focus on the differential effects of the various blood products.

This study is limited by a small and heterogeneous patient population. Thereby, some of the effects may be caused by chance or by regression to the mean. Findings need to be confirmed in a larger sample. The strengths of this study are the use of a fixed dose of FFP and the timing of blood draws both prior and after transfusion, limiting a possible effect of confounders on findings. Thereby, long-term effects of FFP were not investigated in this design.

## Conclusions

In conclusion, this study is the first to describe the effect of a fixed dose of FFP transfusion in critically ill patients. Results suggest that FFP stabilizes endothelial condition.

## Key messages

- Transfusion of fresh frozen plasma in critically ill patients did not aggravate their inflammatory response.
- In critically ill patients, fresh frozen plasma may stabilize endothelial condition.

## Abbreviations

ADAMTS13: a disintegrin and metalloproteinase with a thrombospondin type 1 motif, member 13
APACHE IV score: acute physiology and chronic health evaluation IV score
DIC: disseminated intravascular coagulation
ELISA: enzyme-linked immunosorbent assay
FFP: fresh frozen plasma
ICU: intensive care unit
IL-1: interleukin 1
IL-10: interleukin 10
IL-1RA: interleukin 1 receptor antagonist
IL-1β: interleukin 1 beta
IL-8: interleukin 8
INR: international normalized ratio
IQR: interquartile range
ISTH: International Society on Thrombosis and Haemostasis
LMWH: low-molecular-weight heparin
MCP-1: monocyte chemotactic protein 1
MIP-1A: macrophage inflammatory proteins 1A
sCD40L: soluble CD40 ligand
SD: standard deviation
SOFA score: sequential organ failure assessment score
TNF-α: tumor necrosis factor alpha
TRALI: transfusion-related acute lung injury
vWF: von Willebrand factor
vWF:Ag: von Willebrand factor antigen
